# Making data for good better

**DOI:** 10.1371/journal.pdig.0000010

**Published:** 2022-01-18

**Authors:** Caroline Buckee, Satchit Balsari, Andrew Schroeder

**Affiliations:** 1 Department of Epidemiology, Harvard TH Chan School of Public Health, Boston, Massachusetts, United States of America; 2 Department of Emergency Medicine, Beth Israel Deaconess Medical Center / Harvard Medical School, Boston, Massachusetts, MA, United States of America; 3 Department of Global Health and Population, Harvard TH Chan School of Public Health, Boston, Massachusetts, MA, United States of America; 4 Direct Relief, Santa Barbara, California, United States of America; Massachusetts Institute of Technology, UNITED STATES

Today’s societies produce vast—and increasing—amounts of digital data “exhaust” from daily human activities such as the use of mobile devices, wearables and home sensors; store purchases; and online engagement on social media. Such data have historically been used by corporations to sell products and make life more convenient (even if in unevenly distributed ways), and in limited academic circles, to solve public heath challenges. During the COVID-19 pandemic, however, technology companies started making aggregated human mobility datasets widely available, as part of their corporate social responsibility efforts or “data for good” programs.[1,2] Many companies, researchers, and policy makers unfamiliar with the academic literature realized—for the first time—the potential use of digital data from mobile phones to monitor social distancing and other emergency public health measures.[3] An avalanche of social distancing dashboards, prediction tools, heat-maps, digital contact tracing programs, and symptom-based COVID-19 prediction apps, followed. Despite the long standing excitement about the potential for digital tools, Big Data and AI to transform our lives, these innovations–with some exceptions–have so far had little impact on the greatest public health emergency of our time.[4,5]

Attempts to use digital data streams to rapidly produce public health insights that were not only relevant for local contexts in cities and countries around the world, but also available to decision makers who needed them, exposed enormous gaps across the translational pipeline. The insights from novel data streams which could help drive precise, impactful health programs, and bring effective aid to communities, found limited use among public health and emergency response systems.[6] We share here our experience from the COVID-19 Mobility Data Network (CMDN), now Crisis Ready (crisisready.io), a global collaboration of researchers, mostly infectious disease epidemiologists and data scientists, who served as trusted intermediaries between technology companies willing to share vast amounts of digital data, and policy makers, struggling to incorporate insights from these novel data streams into their decision making.[7] Through our experience with the Network, and using human mobility data as an illustrative example, we recognize three sets of barriers to the successful application of large digital datasets for public good.

First, in the absence of pre-established working relationships with technology companies and data brokers, the data remain primarily confined within private circuits of ownership and control. During the pandemic, data sharing agreements between large technology companies and researchers were hastily cobbled together, often without the right kind of domain expertise in the mix.[8] Second, the lack of standardization, interoperability and information on the uncertainty and biases associated with these data, necessitated complex analytical processing by highly specialized domain experts.[9,10] And finally, local public health departments, understandably unfamiliar with these novel data streams, had neither the bandwidth nor the expertise to sift noise from signal. Ultimately, most efforts did not yield consistently useful information for decision making, particularly in low resource settings, where capacity limitations in the public sector are most acute.[11,12]

We remain hopeful that the vast data sets that people generate every day will be extraordinarily useful for crisis response. For example, data on population mobility provide critical information about population displacement and travel patterns. Satellite imagery and power outage data can help estimate, in near real time, infrastructure disruption. Data from electronic medical records, pharmacies and insurance companies can help map where the medically vulnerable are, what real-time bed capacity is, and what the needs of evacuating communities look like, so that host communities and health systems can better prepare for receiving populations in distress. While we contend that many of the efforts to harness digital data for COVID-19 response did not meet their goals, we also believe that the pandemic expanded awareness of, and access to, novel data streams to a broad range of researchers and policy makers, and are likely to become routinely used for public health in the future.[5]

What would it take for the “Data for Good” agenda to achieve its promised benefits for communities impacted by crises? Substantial work is needed along the entire translational pipeline to understand which data streams and methodologies are most helpful for different response efforts. Most digital data, unless collected on a specific app, are not generated for public health purposes. In order to use them for disaster response the data sharing arrangements between data providers, academic partners, and public health agencies must be in place prior to the disaster.[13] The important privacy concerns raised by the use of individually identifiable digital data necessitate aggregation and anonymization–proprietary processes that often dilute the epidemiological or clinical utility of the data. There is therefore need to standardize these approaches across industries, or for end-users to at least be familiar with the methodology in advance of acute disasters, so as to be able to efficiently combine data from multiple sources. In the absence of existing regulatory frameworks supporting the ethical use of personal data, corporations, researchers and policy makers need to be incentivized to use the data responsibly to derive meaningful insights. The processed data and analysis must finally be shared in near real-time, in the right format, with the right people.[14] Most of these requirements are lacking in public health contexts.

The CMDN’s focus was on the use of digital human mobility data to provide insights into the impact of social distancing, lockdown, and travel restriction policies. Every evening for several months, our researchers shared daily updates from analysis using aggregated population mobility data from social media platforms and telecom companies with local public health partners, explaining the utility and limits of the analysis, and learning what decision makers needed.[15] Perhaps our most important lesson learned from this experience was that translational impact relies on distributed capacity and trusted partnerships.

Currently, the possibilities for improving disaster response with new data and advanced analytics vastly outstrip the ability of most disaster response agencies to employ them. In the wake of 2020, there have been calls for increasing the number of individuals who are trained in epidemiology around the world and can respond to epidemics.[16] We are calling for a similar investment in global technical capacity to translate new data streams into better public health response during crises. Data “bilinguals”, or individuals who have the ability to apply novel digital data to generate specific, contextually relevant, policy-relevant insights, during disasters, will be essential.[17] These individuals may be academics or people working in government agencies or NGOs who have a technical understanding of the appropriate use of digital data streams, and who can work with decision makers on a specific problem. On the policy side, greater sensitization of the potential (and pitfalls) of using these data at various levels of government will facilitate the integration of new data streams into decision making. Data preparedness must become an integral component of disaster preparedness exercises, so data access, methodology and applications are negotiated prior to a disaster.

We conclude that regional collaboration among scientists embedded in or working closely with local response agencies, but supported by a global network of peers, will accelerate the use of these data to help our communities.[18] Without this type of sustained, broad-based and equitably distributed capacity investment, no amount of additional data or improved methodology is likely to result in substantial gains for disaster-affected communities.
